# DNA methylation-based classification of malformations of cortical development in the human brain

**DOI:** 10.1007/s00401-021-02386-0

**Published:** 2021-11-19

**Authors:** Samir Jabari, Katja Kobow, Tom Pieper, Till Hartlieb, Manfred Kudernatsch, Tilman Polster, Christian G. Bien, Thilo Kalbhenn, Matthias Simon, Hajo Hamer, Karl Rössler, Martha Feucht, Angelika Mühlebner, Imad Najm, José Eduardo Peixoto-Santos, Antonio Gil-Nagel, Rafael Toledano Delgado, Angel Aledo-Serrano, Yanghao Hou, Roland Coras, Andreas von Deimling, Ingmar Blümcke

**Affiliations:** 1 Department of Neuropathology, Affiliated Partner of the ERN EpiCARE, Universitätsklinikum Erlangen, Friedrich-Alexander University Erlangen-Nürnberg (FAU), Erlangen, Germany; 2 Department of Neurology, Epilepsy Center, Universitätsklinikum Erlangen, Friedrich-Alexander University Erlangen-Nürnberg (FAU), Erlangen, Germany; 3 Department of Neurosurgery, Universitätsklinikum Erlangen, Friedrich-Alexander University Erlangen-Nürnberg (FAU), Erlangen, Germany; 4 Center for Pediatric Neurology, Neurorehabilitation and Epileptology, Vogtareuth, Germany; 5 Center for Neurosurgery and Epilepsy Surgery, Schön Klinik Vogtareuth, Vogtareuth, Germany; 6 Research Institute, Rehabilitation, Transition, Palliation”, PMU Salzburg, Salzburg, Austria; 7 Department of Epileptology (Krankenhaus Mara), Medical School, Bielefeld University, Bielefeld, Germany; 8 Department of Neurosurgery - Epilepsy Surgery, Evangelisches Klinikum Bethel, Universitätsklinikum OWL, Bielefeld University, Bielefeld, Germany; 9 Department of Neurosurgery, Medical University Vienna, Vienna, Austria; 10 Department of Pediatrics and Adolescent Medicine, Affiliated Partner of the ERN EpiCARE, Medical University Vienna, Vienna, Austria; 11 Department of Pathology, University Medical Center Utrecht, Utrecht, The Netherlands; 12 Department of (Neuro) Pathology, Amsterdam UMC, Location AMC, Amsterdam, The Netherlands; 13 Charles Shor Epilepsy Center, Cleveland Clinic, Cleveland, OH USA; 14 Department of Neurology, Cleveland Clinic, Cleveland, OH USA; 15 Department of Neurology and Neurosurgery, Paulista Medical School, UNIFESP, Sao Paulo, Brazil; 16 Epilepsy Program, Hospital Ruber Internacional, Madrid, Spain; 17 Department of Neuropathology, German Cancer Research Center (DKFZ), Universitätsklinikum Heidelberg, and CCU Neuropathology, Heidelberg, Germany; 18 Institute of Pathology and Southwest Cancer Center, Southwest Hospital, Third Military Medical University (Army Medical University), Chongqing, China

**Keywords:** Brain development, Cortical malformation, Epilepsy, Epigenetic, Deep learning

## Abstract

Malformations of cortical development (MCD) comprise a broad spectrum of structural brain lesions frequently associated with epilepsy. Disease definition and diagnosis remain challenging and are often prone to arbitrary judgment. Molecular classification of histopathological entities may help rationalize the diagnostic process. We present a retrospective, multi-center analysis of genome-wide DNA methylation from human brain specimens obtained from epilepsy surgery using EPIC 850 K BeadChip arrays. A total of 308 samples were included in the study. In the reference cohort, 239 formalin-fixed and paraffin-embedded (FFPE) tissue samples were histopathologically classified as MCD, including 12 major subtype pathologies. They were compared to 15 FFPE samples from surgical non-MCD cortices and 11 FFPE samples from post-mortem non-epilepsy controls. We applied three different statistical approaches to decipher the DNA methylation pattern of histopathological MCD entities, i.e., pairwise comparison, machine learning, and deep learning algorithms. Our deep learning model, which represented a shallow neuronal network, achieved the highest level of accuracy. A test cohort of 43 independent surgical samples from different epilepsy centers was used to test the precision of our DNA methylation-based MCD classifier. All samples from the test cohort were accurately assigned to their disease classes by the algorithm. These data demonstrate DNA methylation-based MCD classification suitability across major histopathological entities amenable to epilepsy surgery and age groups and will help establish an integrated diagnostic classification scheme for epilepsy-associated MCD.

**Supplementary Information:**

The online version contains supplementary material available at 10.1007/s00401-021-02386-0.

## Introduction

Human brain malformations present with a broad spectrum of anatomo-pathological lesions [1], genetic alterations [26], and clinical phenotypes [53]. If the neocortical mantle is affected, a structural lesion is usually classified as malformation of cortical development (MCD), with focal epilepsy being a frequent clinical symptom [26]. Many patients with MCD and focal epilepsy do not respond to anti-seizure medication. However, epilepsy surgery can be a curative treatment option [11, 42]. Recent studies in surgically resected human brain tissue demonstrated that MCD often result from prenatally acquired brain somatic mutations with or without additional germline mutation in developmental signaling pathways governing neuroepithelial proliferation, migration, and cell lineage differentiation [3, 43, 58]. The anatomo-pathological phenotype is likely dependent on the timing of the acquired brain somatic mutation, the targeted cell lineage, and the affected gene [22]. Disease definition and diagnosis of MCD remain challenging in everyday clinical practice. If surgical treatment is suggested, a definitive diagnosis of MCD should be established by histopathology review. Diagnostic terms for MCD categories and subtypes are often defined imprecisely, and histopathological criteria are prone to arbitrary judgment [10, 53]. Previous studies have reported substantial inter- and intra-observer variability in the histopathological diagnosis of MCD, for example, in Focal Cortical Dysplasia (FCD) [10, 52]. The introduction of genetic biomarkers is a growing field but available only for a subgroup of MCD entities so far, i.e., FCD type 2 [2–4, 21, 22, 33, 50, 58] or mild malformations of cortical development with oligodendroglial hyperplasia (MOGHE) [14, 61]. Diagnostic discordance and uncertainty may confound the assignment of genetic variants to disease entities [14] and compromise decision-making in clinical practice as well as the interpretation and validity of clinical observations and trials.

Herein, we address DNA methylation as an objective molecular diagnostic biomarker that can be reliably detected and analyzed from archival human brain FFPE tissue [16, 60, 66]. The methylome in surgical brain tissue represents a combination of both somatically acquired DNA methylation changes, characteristics that reflect the cellular composition of the tissue as well as molecular memory marks in response to environmental or pathogenic cues, including seizures [23, 36–40]. DNA methylation profiling is highly robust and reproducible even from small samples and archival tissue, and such profiles have been widely used to classify CNS tumors successfully [16, 60]. Based on our previous work within single MCD entities [38, 40] (Holthausen et al. accepted in Epilepsia), we developed a comprehensive approach toward the DNA methylation-based classification of major MCD entities across all age groups.

## Materials and methods

### Reference cohort

We reviewed clinical, and MRI data of individuals who underwent surgery for the treatment of their focal pharmaco-resistant epilepsy and were diagnosed with FCD type 1A (*n* = 12), FCD type 2A (*n* = 29), FCD type 2B (*n* = 29), FCD type 3A (*n* = 14), FCD type 3B (*n* = 15, all with ganglioglioma), FCD type 3C (*n* = 17, six with Sturge–Weber Syndrome, four with arterio-venous malformations, and seven with cavernoma), FCD type 3D (*n* = 15, one with traumatic brain injury, five with Rasmussen encephalitis, four with perinatal stroke, and five not further specified), hemimegalencephaly (HME, *n* = 6), mild malformation of cortical development (mMCD, *n* = 28), mMCD with oligodendroglial hyperplasia in epilepsy (MOGHE, *n* = 22), polymicrogyria (PMG, *n* = 33), cortical tuber of tuberous sclerosis complex (TSC, *n* = 19), or temporal lobe epilepsy (TLE, *n* = 15). Based on MRI and histology, all 15 TLE patients were diagnosed with hippocampal sclerosis, but we used only histologically normal temporal neocortex. All cases included into this study have been extensively studied at the microscopic level with Hematoyxlin–Eosin and Cresyl Violet – Luxol Fast Blue stainings available from all FFPE surgical tissue blocks. An immunohistochemistry panel of antibodies recommended for the neuropathology work-up of epilepsy surgery specimens [7, 9], including NeuN, MAP2, GFAP, Vimentin, neurofilament SMI32, Ki67, OLIG2, CD34, CD68 and CD45 epitopes were also made available for each case. Each diagnosis was finally agreed upon consensus by two of our coauthors (IB and RC) applying the International League Against Epilepsy (ILAE) classification system of 2011 [13] and 2013 [12]. An FFPE block containing a prototypic area of the lesion was selected for further processing. Four non-epilepsy autopsy control cases with no known neurological history were also included in the study. From some of these autopsy cases, temporal and frontal neocortex with micro-dissected gray and white matter were sampled and analyzed independently (CTRL, *n* = 11; Table 1, Supplement Table 1, online resource). We obtained written informed consent for molecular genetic investigations and publication of the results from all participating patients or their legal guardians. The Ethics Committee of the Medical Faculty of the Friedrich-Alexander-University (FAU) Erlangen-Nürnberg, Germany, approved this study within the framework of the EU project “DESIRE” (FP7, grant agreement #602,531; AZ 92_14B) and European Reference Network EpiCARE” (grant agreement #769,051; AZ 193_18B).

**Table 1:** Clinical summary of the reference cohort

Diagnosis	*n*	∅ age at surgery	∅ age at onset	∅ duration	Sex (female/male)
FCD 1A	12	9.3 (± 4.6)	2.2 (± 3.1)	6.8 (± 4.3)	7/5
FCD 2A	29	14.7 (± 11.5)	3.5 (± 4.1)	11.2 (± 10.2)	13/16
FCD 2B	29	18.5 (± 14.6)	3.6 (± 4.0)	14.5 (± 12.2)	13/16
FCD 3A	14	45.0 (± 19.7)	9.4 (± 11.2)	31.3 (± 21.0)	9/5
FCD 3B	11(15)	34.5 (± 14.7)	26.6 (± 16.7)	7.91 (± 8.8)	6/5
FCD 3C	17	23.5 (± 18.9)	15.1 (± 14.6)	7.6 (± 8.9)	6/11
FCD 3D	15	15.4 (± 11.0)	5.7 (± 8.4)	6.8 (± 5.4)	7/8
PMG	33	8.5 (± 9.1)	1.6 (± 2.7)	5.9 (± 6.4)	10/23
HME	6	1.3 (± 0.5)	0.1 (± 0.2)	1.3 (± 0.6)	2/4
TSC	19	5.5 (± 6.9)	0 (± 0)	5.5 (± 6.9)	8/11
mMCD	28	24.6 (± 17.6)	9.6 (± 12.3)	11.6 (± 13.3)	13/15
MOGHE	22	8.0 (± 7.2)	8 (± 7.3)	6.0 (± 5.8)	8/12
TLE	15	37.0 (± 15.3)	7.4 (± 7.9)	29.6 (± 16.6)	8/7
CTRL	4(11)	31.3 (± 22.3)	n.a	n.a	3/1

*CTRL* control, *HME* hemimegalencephaly, *FCD* focal cortical dysplasia, *mMCD* mild malformation of cortical development, *MOGHE* mMCD with oligodendroglial hyperplasia in epilepsy, *PMG* polymicrogyria, *TLE* temporal lobe epilepsy, *TSC* tuberous sclerosis complex, *n.a.* not applicable

### Test cohort

The test cohort included 43 independent retrospective surgical samples, including a series of 18 patients provided through the epilepsy surgery program of the Cleveland Clinic, USA. These cases underwent independent iterative evaluation by 20 neuropathologists from 15 different countries and were previously published in the ILAE FCD agreement trial of histopathology and genetic testing [9]. Another 25 samples were provided through the European Epilepsy Brain Bank (EEBB). A clinical summary of all test samples is provided in Table 2, Supplement Table 1, online resource.

**Table 2:** Clinical summary of the test cohort

Diagnosis	*N*	∅ age at surgery	∅ age at onset	∅ duration	Sex (female/male)
FCD 1A	2	10.5 (± 12.0)	4.5 (± 6.4)	6.0 (± 5.7)	1/1
FCD 2A	6	20.9 (± 18.7)	4.4 (± 3.4)	16.2 (± 15.4)	1/5
FCD 2B	6	22.8 (± 12.5)	6.6 (± 6.1)	16.8 (± 13.1)	0/6
FCD 3A	4	20.3 (± 18.9)	3.8 (± 4.3)	16.5 (± 19.7)	2/2
FCD 3C	6	23.3 (± 18.1)	14.2 (± 12.5)	9.9 (± 8.7)	3/3
FCD 3D	2	27.0 (± 14.1)	2.5 (± 3.5)	24.5 (± 17.7)	0/2
mMCD	5	29.0 (± 13.8)	17.0 (± 8.9)	13.0 (± 3.2)	2/3
MOGHE	5	9.7 (± 10.4)	5.0 (± 8.4)	4.7 (± 2.8)	1/4
TLE	3	37.3 (± 9.9)	17.0 (± 11.4)	20.3 (± 2.9)	0/3
TSC	4	3.6 (± 1.9)	0.0 (± 0.0)	3.6 (± 1.9)	2/2

*FCD* focal cortical dysplasia, *mMCD* mild malformation of cortical development, *MOGHE* mMCD with oligodendroglial hyperplasia in epilepsy, *TLE* temporal lobe epilepsy, *TSC*– tuberous sclerosis complex

### DNA extraction

**Fig. 1:**
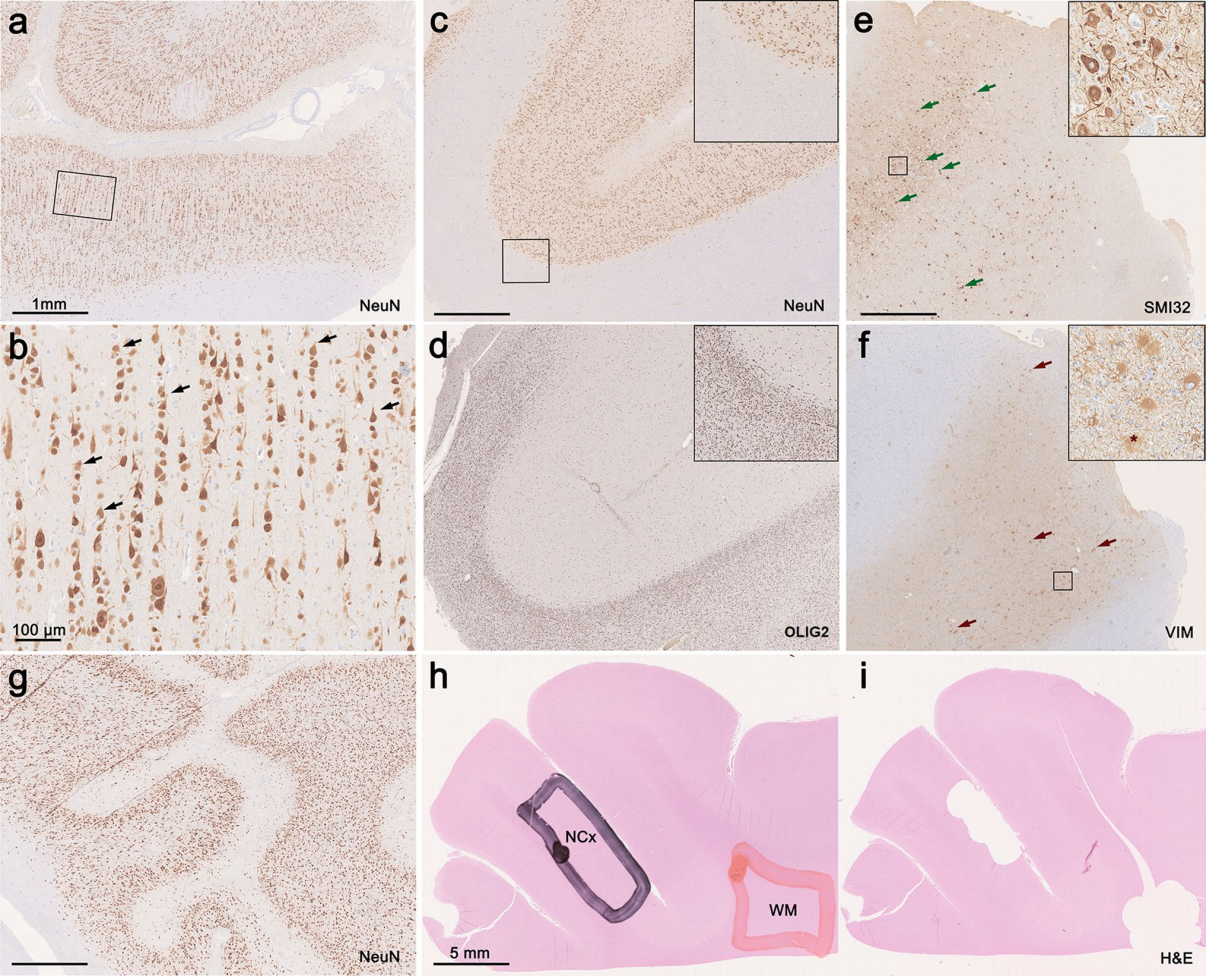
Histopathological findings in representative MCD and control cases from the present cohort. **a**–**b** FCD1A with neocortex showing abundant radial organization of neurons (micro-columns, black arrows, NeuN immunohistochemistry). **c** In MOGHE the cortical ribbon shows no evidence for radial micro-columns or horizontal dyslamination. Instead, gray–white matter blurring with heterotopic neurons subjacent to white matter and (**d**) increase in OLIG2‐immunoreactive oligodendroglial cells are detected. **e** In FCD2B, dysmorphic neurons accumulating non-phosphorylated neurofilament protein and lacking regular anatomic orientation (green arrows, SMI32 immunohistochemistry) as well as balloon cells characterized by large cell bodies occasionally presenting with multiple nuclei (asterisk) positively staining for Vimentin are present (VIM, dark red arrows). **g** In PMG, NeuN immunohistochemistry identifies abnormally folded sulci without pial opening. The cortical ribbon was thinned, mainly four-layered, and the gray to white matter boundaries were blurred with increased numbers of heterotopic neurons in the white matter. (**h**–**i**) Visualization of sampling method: Overview of H&E stained slides depicting selective sampling from regions of interest, e.g., neocortex or white matter, in a control sample. Scale bars are 1 mm if not shown otherwise

A prototypic area within the center of the MCD lesion (neocortex) was identified on H&E slides as described above and macro-dissection performed by punch biopsy (pfm medical, Köln, Germany) or by hand (Fig. 1). DNA was extracted from formalin-fixed paraffin-embedded (FFPE) tissue using the Maxwell 16 FFPE Plus LEV DNA Kit (Promega, Madison, WI, USA), according to the manufacturer’s instructions. DNA concentration was quantified using the Qubit dsDNA BR Assay kit (Invitrogen, Carlsbad, CA, USA).

### Genome-wide DNA methylation profiling and data pre-procession

As described previously, samples were analyzed using Illumina Infinium MethylationEPIC 850 K BeadChip arrays [16, 38]. Briefly, DNA methylation data were generated at the Department of Neuropathology, Universitätsklinikum Heidelberg, Germany. We performed differential DNA methylation analysis using a self-customized Python wrapped cross R package pipeline described in [38] and publicly available at https://github.com/FAU-DLM/Methylr2py. Additionally, we stratified quantile normalized data using the ‘minfi’ ‘preprocessQuantile’ function [25]. After that, probes targeting sex chromosomes, containing single-nucleotide polymorphisms, not uniquely matching, as well as known cross-reactive probes were removed [19]. Consequently, 433,213 CpG probes contained on the EPIC array were used for further analysis. Most significantly differentially methylated CpG between disease entities, as highlighted above, were identified by fitting a regression model with the disease as the target variable using the ‘limma’ R package [59]. All pairwise comparisons between disease groups were identified as contrasts and included in the analysis. Surrogate variable adjustments found one surrogate variable, which we corrected for (‘sva’ R package) [44]. We also corrected for the batch number, duration of epilepsy, white vs. gray matter, and the region the specimen originated from (‘removeBatchEffect’ from ‘limma’). These factors were identified by calculating and plotting the Pearson’s r coefficient as a correlation matrix for all variables of the first six principal components deriving from the corresponding *M* values (data not shown). Then unsupervised dimensionality reduction for cluster analysis on the data was performed. Uniform Manifold Approximation and Projection (UMAP) for general non-linear dimensionality reduction was used for visualization [49]. The following non-default parameters were used: init = random , min_dist = 0.0, spread = 3.0. To confirm the identified clusters from the previous step, we applied unsupervised learning using HDBSCAN as a clustering algorithm [48]. The following non-default parameters were used: min_samples = 1, min_cluster_size = 4. We then re-ran the HDBSCAN algorithm within a loop while randomly down-sampling the data to five samples per disease group for a total of 100 times. We performed additional hierarchical cluster analysis of the corresponding normalized *M* values using python-based seaborns, clustermap plotting method [65]. The following non-default parameters were used: standard_scale = 1, method = ’ward’.

### Machine and deep learning

‘Scikit-learn’, ‘fastai’, and ‘Pytorch’ were used as python-based packages to leverage machine and deep learning [30, 55, 56]. We split the processed methylation data from the steps above into an independent training, validation, and test set. Care was taken that disease classes were stratified across the sets evenly. The test set contained 43 fully independent samples that were not used at any model training and validation stage.

### Machine learning

Using ‘Scikit-learn’, various types of, classic machine learning algorithms were spot-checked on their performance on the dataset via stratified fivefold cross-validation. We separately trained an, extra trees classifier, a, nearest neighbor classifier, a, support vector classifier’ and a, ‘random forest classifier. We then modeled via stratified fivefold cross-validation an ensemble stacking model using, vecstack [32]. Stacked Generalization or “Stacking” is a two-step approach. The first step is to train base machine learning models on the dataset. Therefore, we used the same models as described above. The second step consists of training, a so-called meta-model on the predictions of the base models. This meta-model thereby tries to combine the base models predictions more robustly and accurately. We used the, XGBClassifier from, xgboost as our meta-model [18]. XGBoost stands for “Extreme Gradient Boosting” and is an implementation of gradient boosted decision trees. Boosting is an ensemble technique where new models are added to correct the errors made by existing models. Models are added sequentially until no further improvements can be made. Gradient boosting is an approach where new models are created that predict the residuals or errors of prior models and then added together to make the final prediction. It is called gradient boosting because it uses a gradient descent algorithm to minimize the loss when adding new models [47].

### Deep learning

Utilizing, fastais tabular-learner, we modeled a deep linear neuronal network consisting of three subsequent layers. The first layer contained 500, the second layer 250, and the last layer only one neuron. In a stratified fivefold cross-validation manner, we then trained neuronal networks with a batch size of 32 by cycling the learning rate between 0.0001 and 0.08 for a total of four epochs which was identified by early stopping. Conventionally, the learning rate is decreased as the learning starts converging with time. It is helpful to oscillate the learning rate toward a higher learning rate as it may help get out of saddle points. This method was found to be most efficient in training neuronal networks [5, 63]. To be consistent with plots of the disease clusters, we transformed the predictions of the machine learning and neuronal network models into a two-dimensional space via UMAP dimensionality reduction.

### Classifier performance measures

The performance of the resulting classifier predictions generated by the cross-validation for machine and deep learning models was evaluated by the balanced accuracy, precision, recall, F1 score, and the multiclass area under the receiver operating characteristic (ROC) curve (AUC). Results were plotted into a normalized confusion matrix. The balanced accuracy takes class imbalances into account. Precision and recall were chosen as additional metrics to measure how good samples were classified concerning the fraction of correctly and incorrectly classified samples. Precision is also known as the positive predictive value, and recall is also known as sensitivity. To easier assess these metrics during the training process, we additionally captured the harmonic mean between these scores: the F1 score.

## Results

### Methylation clusters define MCD and non-MCD

To establish a comprehensive MCD reference cohort, we generated genome-wide DNA methylation profiles using Infinium HumanMethylation850K BeadChip arrays (average group size 19; range 6–33 samples) from 239 surgical cases representing the majority of MCD disease entities (FCD 1A, 2A, 2B, 3A, 3B, 3C, 3D, PMG, HME, TSC, mMCD, MOGHE). We also included the intact temporal neocortex of 15 non-MCD TLE patients. Furthermore, we selected 11 samples from 4 autopsy cases representing non-neoplastic, non-MCD, non-epilepsy controls (CTRL; micro-dissected white matter and neocortex were studied individually, Fig. 1). Altogether, this resulted in a combined reference cohort of 265 samples (Table 1).

**Fig. 2:**
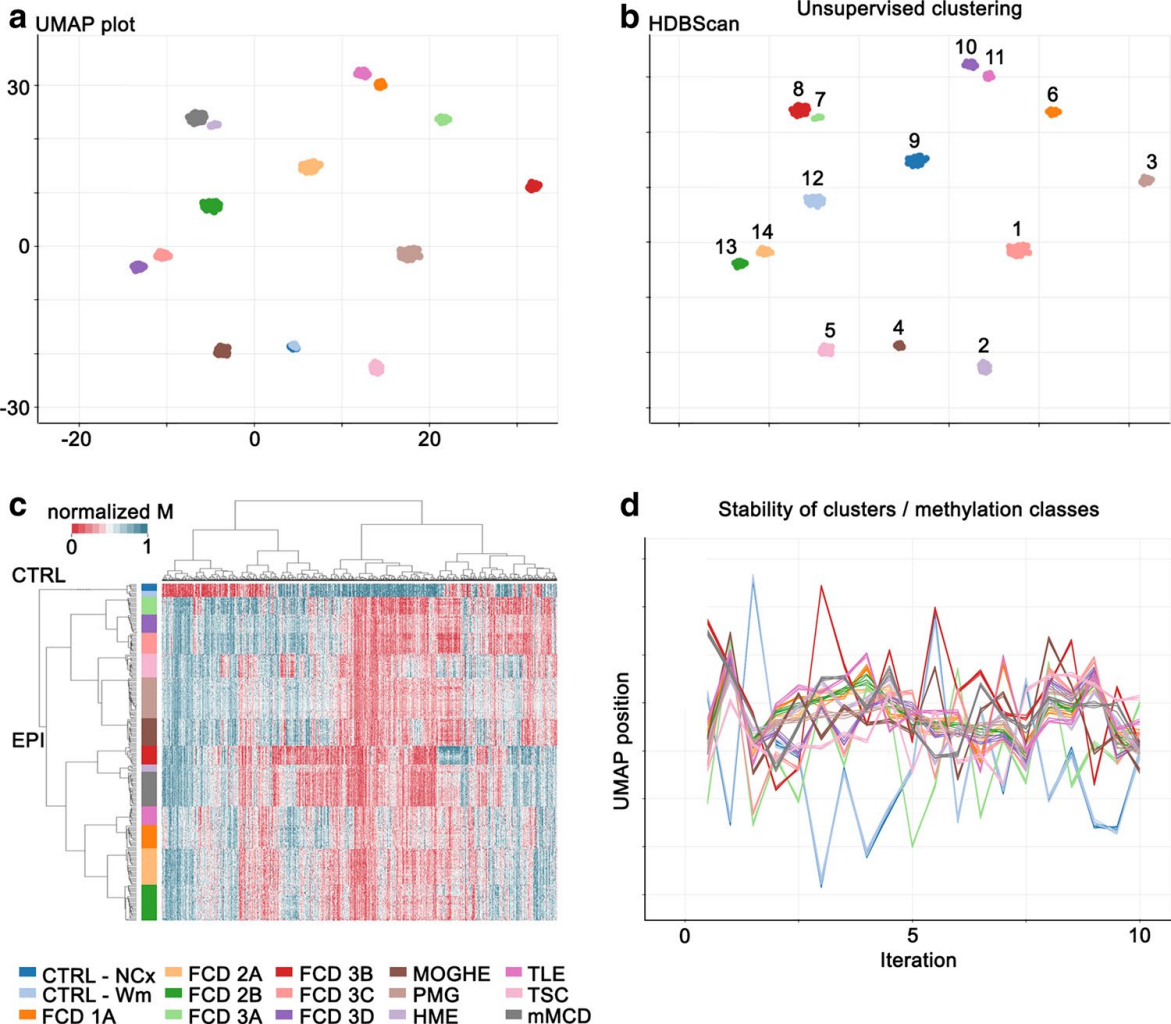
Major MCD subtypes can be distinguished by their DNA methylation profiles. **a** UMAP plot for dimensionality reduction summarizing 12 MCD together with the TLE and control methylation classes of the reference cohort. Methylation classes reflect disease groups based on histology and are color-coded. **b** Confirmatory unsupervised identification of 14 clusters using HDBScan clustering algorithm (independent colors). This approach identified WM and NCx controls as a single uniform cluster. **c** Hierarchical cluster analysis summarizing DNA methylation profiles of 265 samples of the reference cohort. **d** X and Y coordinates of the first 10 of a total of 100 iterations of UMAP dimensionality reduction generated by random down-sampling to assess clustering stability. A line connects axis positions of individual cases. The depiction illustrates the proximity of cases of the same class across iterations, indicative of the high stability of methylation classes independent of the exact composition of the reference cohort. The color scheme for histopathological entities applies to **1–3**, except 1b)

We performed unsupervised dimensionality reduction and hierarchical cluster analysis using 433,213 CpG probes. All disease groups in our analysis formed separate clusters in the UMAP dimensionality reduction characterized by distinct DNA methylation profiles (Fig. 2a). No confounding correlation with any other variable of our data was detected (e.g., sex, age at onset, age at surgery, lobe, neuronal proportion; Supplement Fig. 1, online resource). Unsupervised learning using HDBSCAN as a clustering algorithm (Fig. 2b) and hierarchical cluster analysis confirmed the separation of all samples at the disease level (Fig. 2c). To test the stability of identified clusters, we re-ran the HDBSCAN algorithm within a loop while randomly down-sampling the data to five samples per disease group for a total of 100 times. Thereby we demonstrated that the proximity of cases of the same class was preserved across iterations, indicative of high stability of methylation classes independent of the exact composition of the reference cohort (Fig. 2d).

### Machine and deep learning can distinguish between histopathological entities

**Fig. 3:**
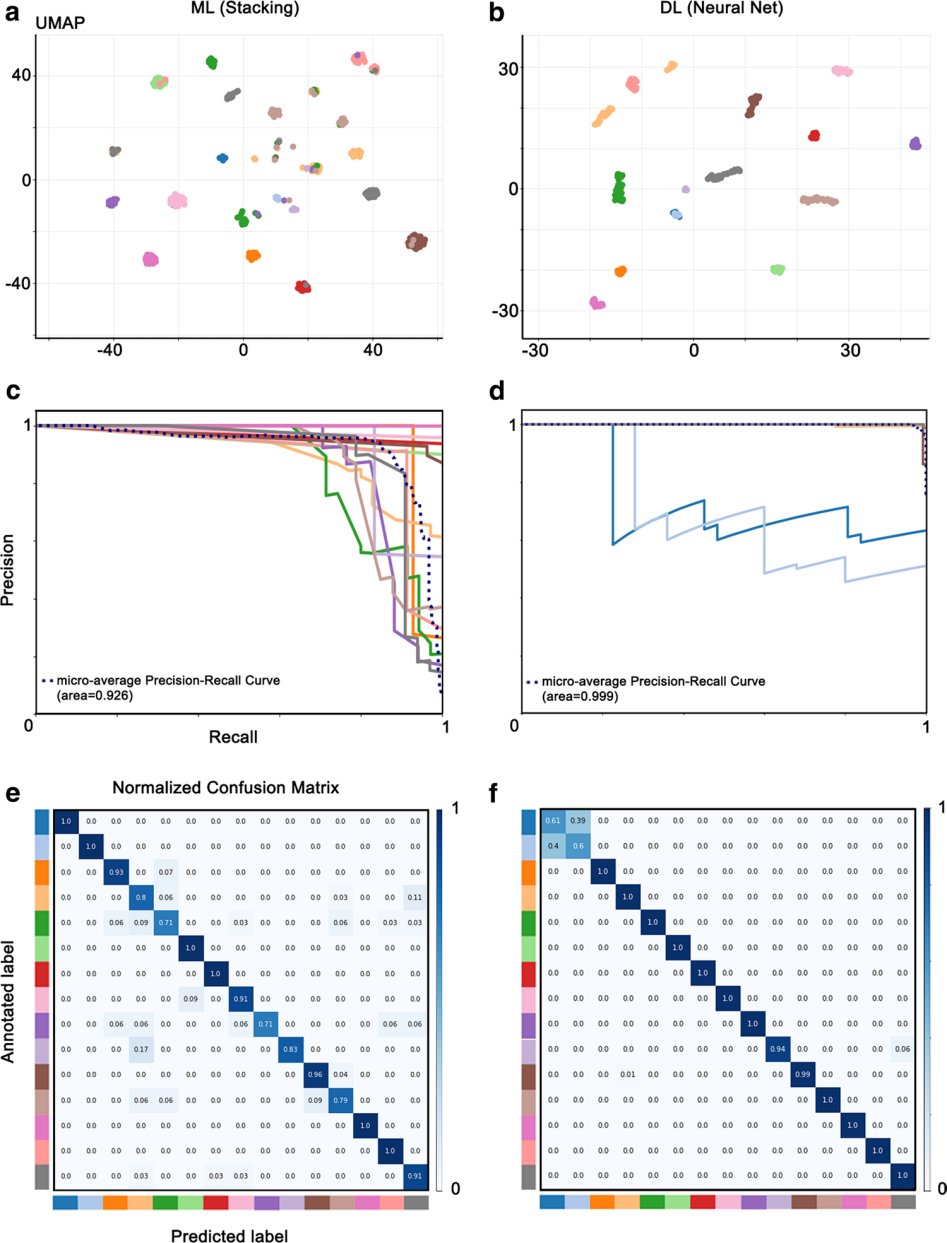
Machine and deep learning models can be trained to distinguish disease entities based on DNA methylation. **a**, **b** UMAP plots showing methylation classes based on ML and DL models. **c**, **d** Precision-recall curve to quantify the efficiency of our multiclass prediction task by ML and DL models. **e**, **f** Performance of the ML and DL models to discriminate 14 classes from the validation and test datasets presented as normalized confusion matrices. The vertical axis indicates the true (annotated) disease class of a sample, and the horizontal axis represents the predicted class. Precision of the DL model is almost 100% in all classes except controls, where, after correction for neuronal proportion, *CTRL* NCx (dark blue) and CTRL—Wm (light blue) form a single methylation class. Color scheme as in Fig. 1. *DL* deep learning; ML – machine learning; *UMAP* uniform manifold approximation and projection

Future application in routine diagnostics requires fast, reproducible, and unbiased classification of samples. It also needs a measure of confidence for the specific call. We trained a, stacking machine learning algorithm (ML), a so-called ensemble method that combines the predictions of several ‘weak’ classifiers to improve prediction accuracy, and compared it to a three-layer shallow neuronal network (i.e., deep learning model, DL). Both ML and DL classifiers raw predictions were UMAP reduced to two dimensions, plotted, and showed excellent methylation class separation (Fig. 3a, b). When running fivefold cross-validation, the machine learning approach reached a balanced classification accuracy of 0.80, positive predictive value (i.e., precision) of 0.73, and sensitivity (i.e., recall) of 0.71, indicating already a good discriminating power (Fig. 3c). However, the neuronal network approach outperformed the ML classifier on all metrics (balanced accuracy 0.94, positive predictive value of 0.98, and sensitivity of 0.98; Fig. 3d). Looking at the confidence of the classification decision for each sample, misclassified samples showed reduced confidence percentage scores for the machine learning and neuronal network approach (Fig. 3e, f), indicating that thresholding the classification confidence might be an appropriate method to minimize the method’ error rate. Using Receiver Operating Characteristic (ROC) curve analysis, we devised an optimal threshold of ≥ 0.9 (Supplement Fig. 2, online resource).

Taken together, our findings provide evidence that methylation profiles are distinct for different epilepsy-associated disease entities and can be discriminated by machine and deep learning methods, which may help to rationalize disease classification and patient stratification.

### Disease classification in an independent test cohort

**Fig. 4:**
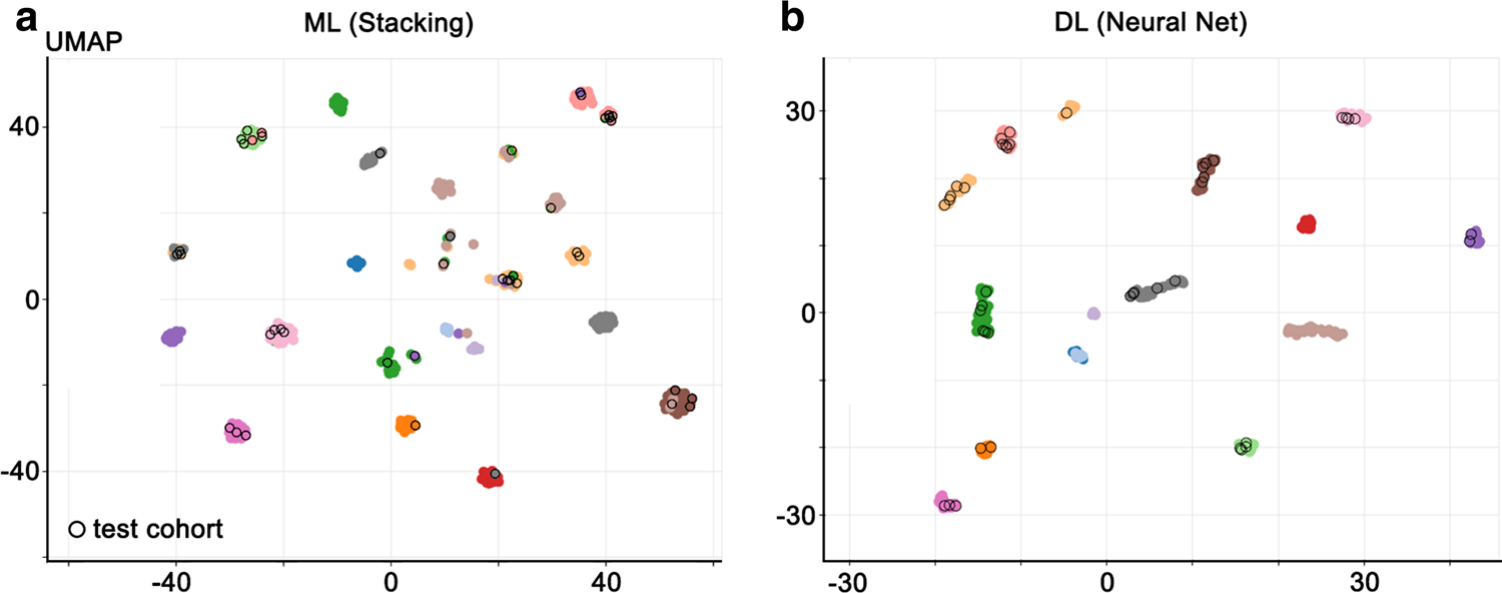
Model testing with independent samples. Mapping of independent test cohort (black circle) to methylation classes identified by our (**a**) ML and (**b**) DL models. Only in the DL model were fully concordant results by pathology and DNA methylation profiling obtained

Next, we tested both models against an independent test cohort (*n* = 43), including 18 samples obtained from the most recent ILAE FCD agreement trial [9]. These difficult-to-classify surgical brain samples obtained from pediatric and adult focal epilepsy patients had undergone multiple rounds of histopathological evaluations by 13 international expert neuropathologists to achieve an agreement on the diagnosis and were now analyzed for DNA methylation. Clinical data for the entire reference cohort are summarized in Table 2 and Supplement table 1, online resource. Methylation profiling and data analysis were performed as for the reference cohort, and test cohort cases were assigned as either ‘matching to a defined DNA methylation class’ (score ≥ 0.9) or as ‘no match’ cases (highest score < 0.9). All profiled samples of the test cohort matched to an established DNA methylation class in both ML (Fig. 4a) and DL models with a classifier score ≥ 0.9 (Fig. 4b). However, only in the DL model were the results obtained by pathology and DNA methylation profiling concordant.

## Discussion

Array-based DNA methylation profiling of formalin-fixed and paraffin-embedded human tissue samples has become a valuable tool to inform histopathology diagnosis in brain tumors [15, 16, 35, 60, 67]. Our data now suggest a practical application also in the diagnostic arena of epilepsy surgery and difficult-to-diagnose brain malformations. We studied a series of 308 cases with pharmaco-resistant epilepsy that underwent surgical treatment and were diagnosed with histopathologically confirmed MCD. This cohort covered the 12 most common MCD subtypes [11] and also our control categories of non-MCD epilepsy and non-epilepsy post-mortems. We demonstrated that DNA methylation profiling distinguished epilepsy tissues from controls and specifically separated all 12 MCD subtypes. These pathology-associated methylation classes could be further discriminated by machine and deep learning algorithms.

Histopathology diagnosis of epilepsy surgery brain tissue poses a particular challenge to everyday clinical practice, especially in FCD [13]. This has been demonstrated several times in international agreement trials with kappa values of 0.4968 for mMCD and FCD 1 in one study [17] and 0.7824 in another study testing the ILAE classification scheme of 2011 [20]. The inter-observer agreement may vary from poor (*k* = 0.16) to good (*k* = 0.68) depending on the additional amount of information being available for the neuropathologist, i.e., immunohistochemistry or gene panel analysis [9]. In fact, the FCD classification scheme has been continuously modified and adapted to address this issue [13, 54]. Difficult-to-anticipate anatomical landmarks in not well preserved or presented surgical specimens and loosely described histopathology features remain the major obstacles to date [52]. While immuno-histochemical markers were introduced and recommended in 2016 by an ad hoc Task Force of the ILAE on diagnostic methods [7], it was not yet included in the FCD consensus classification scheme. Moreover, the small number of epilepsy surgery cases in an individual center requires continuous training of the neuropathologist, but only a few opportunities exist to attend specialized training programs [57]. Hence, developing an easy-to-use and FFPE-compatible diagnostic tool is of great importance to enhance the diagnostic yield in MCD, overcome inter-observer variability and standardize MCD diagnostics across centers and clinical trials [9].

DNA methylation analysis already fosters detection and molecular characterization of more specific and new disease entities in the broad group of brain tumors, particularly those characterized by specific pathogenic variants or treatable by targeted therapies [28, 34, 41, 51, 64, 68]. We assume that the disease classification of MCD will also show such a dynamic adaptation, with more molecular genetic data becoming available over time. An integrated phenotype–genotype classification scheme has already been proposed for FCD, mainly Type 2, where brain somatic mutations in MTOR and GATOR signaling have been repeatedly identified [3, 8, 22]. Another practical example is MOGHE, which was first described histopathologically in 2017, specified further by a characteristic MRI signature, and finally revealed brain somatic mutations in the UDP-galactose transporter gene *SLC35A2* [8, 14, 27, 61]. In the present study, MOGHE cases showed a specific DNA methylation cluster, distinct from the clinically most challenging differential diagnosis of FCD 1A (Holthausen et al. accepted in Epilepsia) or other mMCD and non-lesional focal epilepsy [14].

Further, we recently identified polymicrogyria (PMG) with mosaic trisomy of the long arm of chromosome 1 as a molecularly defined MCD subgroup [38]. Its specific DNA methylation signature and copy number profile clinically associated with a unilateral frontal or hemispheric PMG without hemimegalencephaly, a severe form of intractable epilepsy with seizure onset in the first months of life, and severe developmental delay. Thus, it was to represent a distinct subtype within the spectrum of PMG disorders.

Yet another ongoing interest and research area has been low-grade developmental brain tumors associated with early-onset epilepsy, with many new categories implemented in each novel WHO classification scheme [6]. DNA methylation revealed distinct molecular signatures for many of these new brain tumor entities [24, 31, 62], including papillary glioneuronal tumors [29] and, more recently also isomorphic diffuse glioma [66]. However, epilepsy surgery tissue is distinct from CNS tumor samples and imposes specific challenges to be addressed (see Supplement Fig. 3, online resource, for details). First, cortical malformations obtained from epilepsy surgery usually contain low-level mosaicism of affected cells mixed with normally developed neurons and glial cells. MCD also result from pathogenic variants at variable sites of the affected genes compared to more frequent hot-spot mutations in brain tumors. Many MCD pathologies completely lack any known driver mutation. While tumors are considered to develop from single cells of origin by clonal evolution so that all cells within the tumor harbor the same mutation, epileptic tissue fails to show that pattern. Even neighboring neurons in the normal brain may carry a genetic profile much different from each other [45, 46].

In contrast, the genomic DNA methylation in bulk epileptic brain tissue has been highly specific to the seizure phenotype across species and model systems irrespective of cellular composition and appeared further specific for etiology and histopathology [23, 38–40]. While previous studies analyzed only small sample cohorts focusing on specific pathologies, e.g., FCD or PMG, the present study is the first comprehensive description of diagnostically valuable DNA methylation signatures across the broad spectrum of MCD and all age groups. Continuing efforts for molecular characterization of epilepsy surgery tissue may in future enhance our understanding of, e.g., hemimegalencephaly, which remains a solely macroscopic diagnosis based on MRI so far, or other heterogeneous and not yet well-defined diagnostic entities, e.g., FCD Type 1, and non-lesional tissue. The inclusion of new diagnostic MCD entities based on such an advanced molecular diagnostic workup will, however, require a careful review to advance clinical patient management and precision medicine in the arena of epileptology.

## Supplementary Information

Below is the link to the electronic supplementary material.

Supplementary file1 (PDF 1328 KB)

## Data Availability

The complete methylation data required for constructing the classifier, i.e., the reference and the independent test cohorts, have been deposited in NCBI’s Gene Expression Omnibus (GEO, http://www.ncbi.nlm.nih.gov/geo) under accession numbers GSE185090 and GSE156374. Supplement Table 1 provides IDAT-file names and assignments to patient characteristics (excluding previously published samples [38]).
