# Supplementary material for: DNA methylation-based classification of malformations of cortical development in the human brain
Source: Acta Neuropathol. 2021 Nov 19;143(1):93–104. doi: 10.1007/s00401-021-02386-0 (PMC8732912; doi:10.1007/s00401-021-02386-0)
Supplement: Supplementary file 1 — Supplementary file1 (PDF 1328 KB) [file 401_2021_2386_MOESM1_ESM.pdf]

Supplement Figure 1

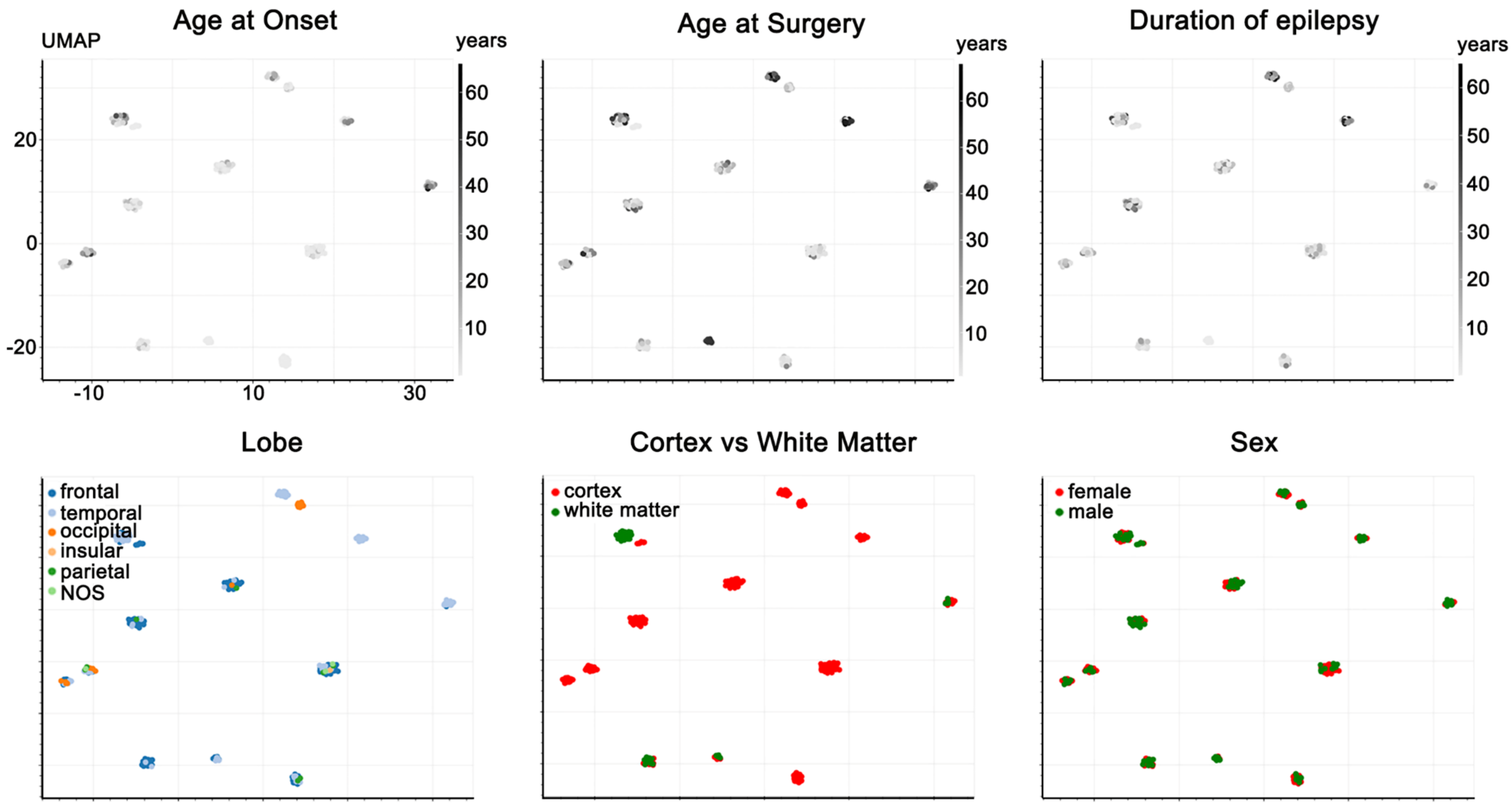

Legend to Supplement Fig. 1: Clustering of samples not driven by the tested covariates. UMAP plots were generated displaying the lack of confounding variable influence. Samples were labeled with the covariates age at onset, age at surgery, duration of epilepsy, sampled lobe (temporal, frontal, parietal, occipital, insular, NOS – not otherwise specified), cortex (green) or white matter (red), and sex: male (green), female (red). The tested covariates did not drive the clustering of samples.

Supplement Figure 2

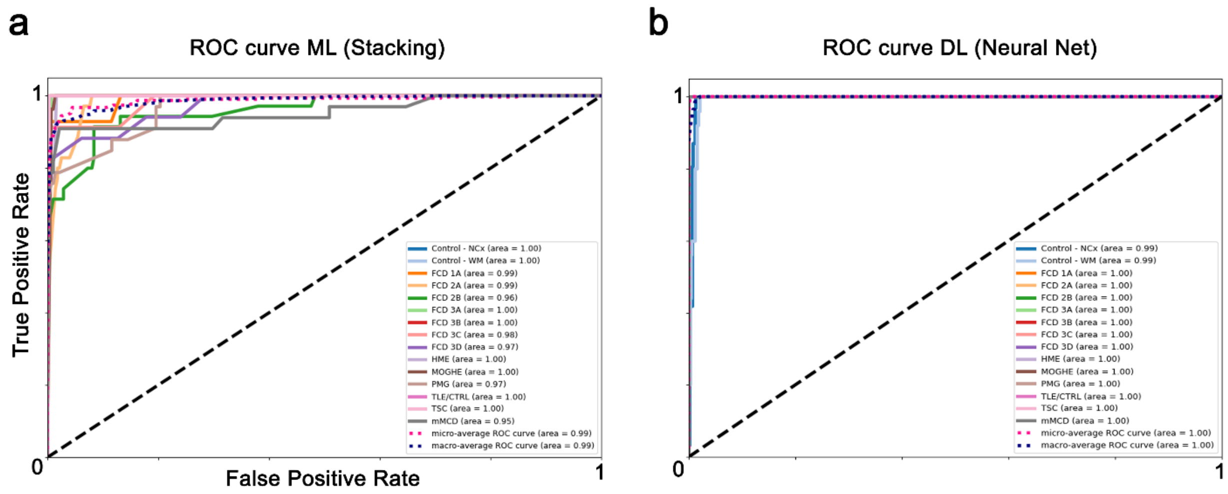

Legend to Supplement Fig. 2: ROC curves summarizing true and false positive rates for assigning a pathology class label based on our machine learning (ML) or deep learning model (DL).

Supplement Figure 3

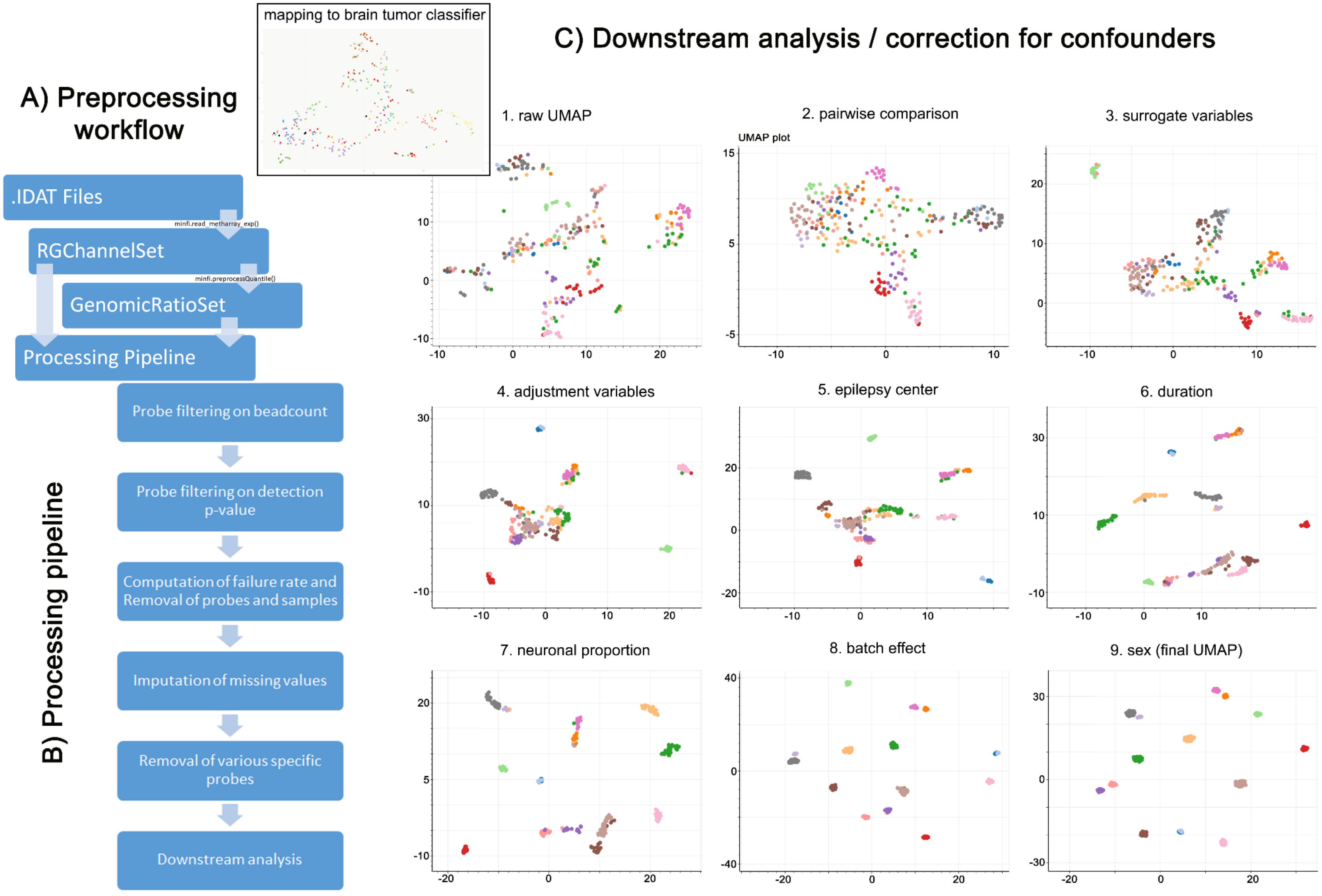

Legend to Supplement Fig. 3: Stepwise pipeline evolution and downstream effects on the MCD classification task. A t-SNE mapping of all samples from the present study into the brain tumor classifier shows no strong separation of methylation classes [16], highlighting the need for a specific pipeline addressing structural brain lesions in epilepsy. The present data analysis pipeline consists of A) preprocessing workflow, B) the processing pipeline, which addressed quality control issues concerning probes and samples, and C) the downstream analysis, which focused on the identification of and correction for confounders. UMAP plots were generated following correction for surrogate, adjustment, and correction variables (e.g., epilepsy center, onset, duration, age at surgery, cellular heterogeneity, batch effects, and sex; panels 1.-9.). For clarity, not all single corrections steps are displayed. Samples were labeled according to their histopathological diagnosis using the same color code as in **Fig. 1**. MCD – malformation of cortical development, UMAP - Uniform Manifold Approximation and Projection, t-SNE - t-distributed stochastic neighbor embedding.

Supplement Table 1

ID	diagnosis	diagnosis+/ ass. lesion	lobe	sex	onset	duration	age at OP	idat	cohort
1	Control/NCx		frontal	M	0.0	0.0	52.0	202939390010_R07C01	Reference
2	Control/NCx		frontal	M	0.0	0.0	52.0	202944920003_R05C01	Reference
3	FCD 1A		occipital	F	0.25	6.75	7.0	202148010052_R08C01	Reference
4	FCD 1A		occipital	M	3.5	3.5	7.0	202148010053_R01C01	Reference
5	FCD 1A		occipital	F	1.5	10.5	12.0	202148010053_R02C01	Reference
6	FCD 1A		occipital	M	3.0	12.0	15.0	202148010053_R03C01	Reference
7	FCD 1A		occipital	F	0.0	3.0	3.0	202148010053_R04C01	Reference
8	FCD 1A		occipital	M	0.9	2.1	3.0	202148010053_R05C01	Reference
9	FCD 1A		occipital	M	0.5	10.5	11.0	202818860046_R04C01	Reference
10	FCD 1A		occipital	F	10.0	8.0	18.0	202818860046_R06C01	Reference
11	FCD 1A		occipital	F	6.0	5.0	11.0	202818860046_R08C01	Reference
12	FCD 1A		occipital	M	0.25	10.75	11.0	202818860053_R02C01	Reference
13	FCD 1A		occipital	F	0.0	0.0	9.0	203726680023_R06C01	Reference
14	FCD 1A		occipital	F	0.0	0.0	5.0	203726680023_R07C01	Reference
15	FCD 2A		frontal	M	10.0	9.0	19.0	202818860117_R07C01	Reference
16	FCD 2A		frontal	M	2.0	9.0	11.0	202827620173_R05C01	Reference
17	FCD 2A		frontal	M	4.0	25.0	29.0	202093110113_R04C01	Reference
18	FCD 2A		frontal	M	4.0	25.0	29.0	202818860117_R08C01	Reference
19	FCD 2A		frontal	M	18.0	22.0	40.0	202827620173_R06C01	Reference
20	FCD 2A		occipital	M	3.0	3.0	6.0	202093110113_R06C01	Reference
21	FCD 2A		temporal	F	0.0	1.0	1.0	202093110113_R05C01	Reference
22	FCD 2A		frontal	F	4.0	13.0	17.0	202822930036_R08C01	Reference
23	FCD 2A		frontal	M	3.5	11.5	15.0	202818860117_R03C01	Reference
24	FCD 2A		frontal	M	0.0	1.0	1.0	202827620173_R07C01	Reference
25	FCD 2A		frontal	F	0.2	10.8	11.0	202827620174_R02C01	Reference
26	FCD 2A		frontal	M	0.1	1.0	2.0	202827620174_R05C01	Reference
27	FCD 2B		frontal	F	4.0	5.0	9.0	202093110113_R07C01	Reference
28	FCD 2B		frontal	F	15.0	17.0	32.0	202148010052_R01C01	Reference
29	FCD 2B		frontal	M	0.0	24.0	24.0	202148010052_R03C01	Reference
30	FCD 2B		frontal	F	0.2	10.8	33.0	202831040056_R02C01	Reference
31	FCD 2B		frontal	M	6.0	19.0	25.0	202822930161_R07C01	Reference
32	FCD 2B		frontal	F	5.0	8.0	13.0	202822930161_R08C01	Reference
33	FCD 2B		frontal	F	3.0	33.0	36.0	202148010052_R04C01	Reference
34	FCD 2B		frontal	F	13.0	29.0	42.0	202831040055_R06C01	Reference
35	FCD 2B		frontal	M	0.0	2.0	2.0	202148010052_R06C01	Reference
36	FCD 2B		frontal	M	0.0	8.0	8.0	202148010052_R07C01	Reference
37	FCD 3A	HS	temporal	F	3.0	44.0	47.0	203197470168_R03C01	Reference
38	FCD 3A	HS	temporal	F	2.0	65.0	67.0	203197470168_R07C01	Reference
39	FCD 3A	HS	temporal	F	3.0	61.0	64.0	203197470168_R08C01	Reference
40	FCD 3A	HS	temporal	F	6.0	49.0	55.0	203197470098_R01C01	Reference
41	FCD 3A	HS	temporal	M	27.0	22.0	49.0	203197470098_R08C01	Reference
42	FCD 3A	HS	temporal	F	25.0	29.0	54.0	203197470168_R04C01	Reference
43	FCD 3A	HS	temporal	F	6.0	52.0	58.0	203197470168_R05C01	Reference
44	FCD 3A	HS	temporal	F	25.0	27.0	52.0	203197470168_R06C01	Reference
45	FCD 3A	HS	temporal	M	2.0	23.0	25.0	203197470098_R03C01	Reference
46	FCD 3A	HS	temporal	M	5.0	30.0	35.0	203197470098_R04C01	Reference
47	FCD 3A	HS	temporal	F	28.0	27.0	55.0	203197470098_R05C01	Reference
48	FCD 3A	HS	temporal	M	0.0	0.0	3.0	203197470098_R06C01	Reference
49	FCD 3A	HS	temporal	M	0.0	9.0	9.0	203197470098_R02C01	Reference
50	FCD 3A	HS	temporal	F	0.0	0.0	57.0	203197470098_R07C01	Reference
51	FCD 3B	GG	temporal	F	3.0	25.0	28.0	203273200108_R07C01	Reference
52	FCD 3B	GG	temporal	F	11.0	0.0	11.0	203273200108_R08C01	Reference
53	FCD 3B	GG	temporal	M	28.0	4.0	32.0	203189480060_R01C01	Reference
54	FCD 3B	GG	temporal	M	20.0	10.0	30.0	203189480060_R02C01	Reference

**55**	FCD 3B	GG	frontal	F	66.0	1.0	67.0	203189480060_R03C01	Reference
**56**	FCD 3B	GG	temporal	M	20.0	3.0	23.0	203189480060_R04C01	Reference
**57**	FCD 3B	GG	temporal	F	21.0	23.0	44.0	203189480060_R05C01	Reference
**58**	FCD 3B	GG	temporal	M	44.0	2.0	46.0	203189480060_R06C01	Reference
**59**	FCD 3B	GG	temporal	F	23.0	3.0	26.0	203189480060_R07C01	Reference
**60**	FCD 3B	GG	temporal	F	27.0	4.0	31.0	203189480060_R08C01	Reference
**61**	FCD 3B	GG	temporal	M	29.0	12.0	41.0	203189480017_R01C01	Reference
**62**	FCD 3B	GG (tumor sampled)	temporal	F	25.0	3.0	28.0	203189480071_R03C01	Reference
**63**	FCD 3B	GG (tumor sampled)	temporal	F	11.0	0.0	11.0	203189480071_R04C01	Reference
**64**	FCD 3B	GG (tumor sampled)	temporal	M	28.0	4.0	32.0	203259190053_R03C01	Reference
**65**	FCD 3B	GG (tumor sampled)	temporal	M	20.0	10.0	30.0	203259190053_R04C01	Reference
**66**	FCD 3C	SWS	occipital	F	5.0	4.0	9.0	203219640200_R05C01	Reference
**67**	FCD 3C	SWS	frontal	F	47.0	1.0	48.0	203197470168_R01C01	Reference
**68**	FCD 3C	CAV	frontal	M	12.0	4.0	16.0	203219640200_R01C01	Reference
**69**	FCD 3C	CAV	temporal	F	30.0	26.0	56.0	203219640200_R02C01	Reference
**70**	FCD 3C	CAV	temporal	M	23.0	30.0	53.0	203219640200_R03C01	Reference
**71**	FCD 3C	AVM	frontal	M	12.0	1.0	13.0	203219640200_R06C01	Reference
**72**	FCD 3C	CAV	temporal	M	28.0	17.0	45.0	203219640218_R06C01	Reference
**73**	FCD 3C	CAV	occipital	M	19.0	9.0	28.0	203219640218_R07C01	Reference
**74**	FCD 3C	CAV	temporal	M	42.0	1.0	43.0	203219640200_R07C01	Reference
**75**	FCD 3C	CAV	occipital	M	15.0	15.0	30.0	203219640200_R08C01	Reference
**76**	FCD 3C	SWS	temporal	F	0.4	2.6	3.0	203219640218_R04C01	Reference
**77**	FCD 3C	SWS	frontal	F	0.5	1.5	2.0	203219640218_R05C01	Reference
**78**	FCD 3C	SWS	parietal	M	2.0	4.2	5.0	203219640218_R08C01	Reference
**79**	FCD 3C	SWS	unknown	M	1.0	0.8	2.0	203219640200_R04C01	Reference
**80**	FCD 3C	AVM	occipital	M	9.4	2.5	12.0	203197470168_R02C01	Reference
**81**	FCD 3C	AVM	frontal	F	9.5	9.5	19.0	203197470213_R08C01	Reference
**82**	FCD 3C	AVM	parietal	M	0.0	0.0	16.0	203197470090_R01C01	Reference
**83**	FCD 3D	stroke	frontal	F	10.0	7.0	17.0	203219730159_R01C01	Reference
**84**	FCD 3D	stroke	frontal	M	0.5	4.5	5.0	203219730159_R02C01	Reference
**85**	FCD 3D	stroke	temporal	M	1.0	1.0	2.0	203219730159_R03C01	Reference
**86**	FCD 3D	RE	temporal	M	7.0	4.0	11.0	203219640118_R07C01	Reference
**87**	FCD 3D	NS	occipital	M	11.0	20.0	31.0	203219730159_R06C01	Reference
**88**	FCD 3D	RE	temporal	F	33.0	9.0	42.0	203219640218_R03C01	Reference
**89**	FCD 3D	NA	occipital	F	0.0	0.0	31.0	203219730159_R05C01	Reference
**90**	FCD 3D	stroke	frontal	F	0.0	0.0	14.0	203219730159_R07C01	Reference
**91**	FCD 3D	NA	frontal	F	3.3	3.7	7.0	203219730055_R08C01	Reference
**92**	FCD 3D	NA	occipital	M	3.0	9.0	12.0	203219730159_R04C01	Reference
**93**	FCD 3D	stroke	occipital	F	0.6	13.4	14.0	203219730159_R08C01	Reference
**94**	FCD 3D	TBI	frontal	M	1.0	7.0	8.0	203219640118_R06C01	Reference
**95**	FCD 3D	RE	frontal	M	3.3	10.7	14.0	203219640118_R08C01	Reference
**96**	FCD 3D	RE	frontal	F	6.0	9.0	15.0	203219640218_R01C01	Reference
**97**	FCD 3D	RE	temporal	M	5.0	3.0	8.0	203219640218_R02C01	Reference
**98**	mMCD		temporal	M	16.0	30.0	46.0	203273200229_R02C01	Reference
**99**	mMCD		temporal	M	2.0	13.0	15.0	203273200229_R04C01	Reference
**100**	mMCD		temporal	F	22.0	3.0	25.0	203273200229_R05C01	Reference
**101**	mMCD		temporal	M	42.0	7.0	49.0	203273200229_R06C01	Reference
**102**	mMCD		temporal	F	23.0	12.0	35.0	203273630036_R02C01	Reference
**103**	mMCD		temporal	M	7.0	17.0	24.0	203219750057_R04C01	Reference
**104**	mMCD		temporal	F	15.0	49.0	64.0	203219750057_R05C01	Reference
**105**	mMCD		temporal	F	3.0	24.0	27.0	203219750057_R06C01	Reference
**106**	mMCD		temporal	F	6.0	44.0	50.0	203219730060_R01C01	Reference
**107**	mMCD		temporal	M	10.0	6.0	16.0	203273200229_R03C01	Reference
**108**	mMCD		temporal	M	27.0	27.0	54.0	203273200229_R07C01	Reference
**109**	mMCD		temporal	F	43.0	6.0	49.0	203273200229_R08C01	Reference

**110**	mMCD		frontal	M	19.0	8.0	27.0	203273630036_R07C01	Reference
**111**	mMCD		temporal	F	0.0	0.0	14.0	203273200231_R08C01	Reference
**112**	mMCD		frontal	M	0.0	0.0	2.0	203273200229_R01C01	Reference
**113**	mMCD		frontal	F	0.0	0.0	38.0	203273630036_R05C01	Reference
**114**	mMCD		frontal	M	0.0	0.0	19.0	203273630036_R06C01	Reference
**115**	mMCD		frontal	M	12.0	20.0	32.0	203273200108_R02C01	Reference
**116**	mMCD		frontal	F	4.0	23.0	27.0	203273200108_R03C01	Reference
**117**	mMCD		frontal	F	0.0	0.0	9.0	203273200108_R05C01	Reference
**118**	mMCD		frontal	F	0.0	0.0	13.0	203273200108_R06C01	Reference
**119**	mMCD		temporal	M	5.0	8.5	13.0	203219750057_R03C01	Reference
**120**	mMCD		temporal	M	2.0	11.0	13.0	203219750057_R07C01	Reference
**121**	mMCD		temporal	F	5.5	7.5	13.0	203219730060_R02C01	Reference
**122**	mMCD		temporal	M	2.5	2.5	5.0	203273630036_R01C01	Reference
**123**	mMCD		temporal	M	0.6	1.9	3.0	203273630036_R03C01	Reference
**124**	mMCD		frontal	F	0.5	2.6	4.0	203273630036_R04C01	Reference
**125**	mMCD		frontal	M	0.9	2.1	3.0	203273200108_R04C01	Reference
**126**	MOGHE		temporal	M	0.0	4.0	4.0	202818860023_R03C01	Reference
**127**	MOGHE		frontal	M	2.6	20.4	23.0	202818860023_R05C01	Reference
**128**	MOGHE		frontal	F	1.2	0.8	2.0	202818860018_R02C01	Reference
**129**	MOGHE		frontal	M	0.5	4.5	5.0	202818860018_R04C01	Reference
**130**	MOGHE		frontal	F	1.0	2.0	3.0	202818860018_R06C01	Reference
**131**	MOGHE		frontal	M	0.0	3.0	3.0	202818860018_R08C01	Reference
**132**	MOGHE		frontal	M	0.0	2.0	2.0	203041550107_R02C01	Reference
**133**	MOGHE		frontal	F	0.3	9.7	10.0	203041550107_R04C01	Reference
**134**	MOGHE		frontal	M	12.0	6.0	18.0	203041550107_R06C01	Reference
**135**	MOGHE		frontal	F	0.0	3.0	3.0	203041550107_R08C01	Reference
**136**	MOGHE		frontal	M	0.3	4.7	15.0	202944920001_R02C01	Reference
**137**	MOGHE		temporal	M	0.0	4.0	4.0	202818860023_R04C01	Reference
**138**	MOGHE		frontal	M	2.6	20.4	23.0	202818860023_R06C01	Reference
**139**	MOGHE		frontal	F	1.2	0.8	2.0	202818860018_R03C01	Reference
**140**	MOGHE		frontal	M	0.5	4.5	5.0	202818860018_R05C01	Reference
**141**	MOGHE		frontal	F	1.0	2.0	3.0	202818860018_R07C01	Reference
**142**	MOGHE		frontal	M	0.0	3.0	3.0	203041550107_R01C01	Reference
**143**	MOGHE		frontal	M	0.0	2.0	2.0	203041550107_R03C01	Reference
**144**	MOGHE		frontal	F	0.0	10.0	10.0	203041550107_R05C01	Reference
**145**	MOGHE		frontal	M	12.0	6.0	18.0	203041550107_R07C01	Reference
**146**	MOGHE		frontal	F	0.0	3.0	3.0	202944920001_R01C01	Reference
**147**	MOGHE		frontal	M	0.0	15.0	15.0	202944920001_R03C01	Reference
**148**	PMG		frontal	M	0.5	2.0	2.5	203511880025_R08C01	Reference
**149**	PMG		frontal	M	1.0	16.0	17.0	203511880026_R01C01	Reference
**150**	PMG		frontal	M	0.0	0.0	32.0	203511880026_R04C01	Reference
**151**	PMG		frontal	M	6.0	22.0	28.0	203726680023_R01C01	Reference
**152**	PMG		unknown	M	0.0	3.0	3.0	203219750116_R01C01	Reference
**153**	PMG		frontal	F	0.0	1.0	1.0	203197470090_R02C01	Reference
**154**	PMG		frontal	F	0.0	1.0	1.0	203197470164_R08C01	Reference
**155**	TSC		parietal	F	0.0	2.0	2.0	202944920001_R04C01	Reference
**156**	TSC		temporal	M	0.0	1.0	1.0	202944920001_R05C01	Reference
**157**	TSC		parietal	F	0.0	5.0	5.0	202944920001_R07C01	Reference
**158**	TSC		temporal	M	0.0	4.0	4.0	202944920001_R08C01	Reference
**159**	TSC		frontal	M	0.0	3.0	3.0	203220070086_R01C01	Reference
**160**	TSC		frontal	M	0.0	2.0	2.0	203220070086_R03C01	Reference
**161**	TSC		frontal	M	0.0	3.0	3.0	203220070086_R04C01	Reference
**162**	TSC		temporal	F	0.0	2.0	2.0	203220070086_R06C01	Reference
**163**	TSC		frontal	M	0.0	1.0	1.0	203220070086_R07C01	Reference
**164**	TSC		frontal	F	0.0	3.0	3.0	203220070086_R08C01	Reference
**165**	TSC		frontal	M	0.0	1.0	1.0	203220070058_R01C01	Reference
**166**	TSC		frontal	M	0.0	1.0	1.0	203220070058_R02C01	Reference

**167**	TSC		frontal	F	0.0	8.0	8.0	202931510113_R07C01	Reference
**168**	TSC		frontal	F	0.0	13.0	13.0	202931510113_R03C01	Reference
**169**	TSC		frontal	M	0.0	29.0	29.0	202944920001_R06C01	Reference
**170**	TSC		temporal	M	0.0	6.0	6.0	203220070086_R02C01	Reference
**171**	TSC		temporal	M	0.0	15.0	15.0	203220070086_R05C01	Reference
**172**	TSC		frontal	F	0.0	1.0	1.0	203220070058_R03C01	Reference
**173**	FCD 1A		NOS	F	0	2	2	205555380023_R03C01	Test/EEBB
**174**	FCD 2A		frontal	M	9	25	36	205555380023_R05C01	Test/EEBB
**175**	FCD 2A		temporal	F	3	2	5	205555380023_R06C01	Test/EEBB
**176**	FCD 2B		parietal	M	0	1	1	205555380023_R07C01	Test/EEBB
**177**	FCD 2B		frontal	M	10	10	20	205555380023_R08C01	Test/EEBB
**178**	FCD 3A		temporal	M	7	24	31	205555380058_R01C01	Test/EEBB
**179**	FCD 3A		temporal	M	0	1	1	205555380058_R02C01	Test/EEBB
**180**	FCD 3A		temporal	F	8	0	8	205555380058_R03C01	Test/EEBB
**181**	FCD 3A		temporal	F	0	41	41	205555380058_R04C01	Test/EEBB
**182**	FCD 3C		temporal	M	24	11	35	205555380058_R05C01	Test/EEBB
**183**	FCD 3C		occipital	M	NA	NA	17	205555380058_R06C01	Test/EEBB
**184**	FCD 3C		occipital	F	1	4	5	205555380058_R07C01	Test/EEBB
**185**	FCD 3C		NOS	M	0,3	1,2	1,5	205555380058_R08C01	Test/EEBB
**186**	FCD 3C		temporal	F	24	9	35	205555380065_R01C01	Test/EEBB
**187**	FCD 3C		temporal	F	22	24	46	205555380065_R02C01	Test/EEBB
**188**	mMCD		temporal	F	15	32	47	205555380065_R07C01	Test/EEBB
**189**	mMCD		temporal	F	21	18	39	205555380065_R08C01	Test/EEBB
**190**	mMCD		NOS	M	2	14	16	205555380121_R02C01	Test/EEBB
**191**	MOGHE		frontal	F	1,5	3,3	4,8	205555380077_R01C01	Test/EEBB
**192**	MOGHE		NOS	M	2	2	4	205555380121_R01C01	Test/EEBB
**193**	MOGHE		NOS	M	1	2	3	205555380121_R03C01	Test/EEBB
**194**	TSC		NOS	F	0	6	6	205555380121_R04C01	Test/EEBB
**195**	TSC		frontal	F	0	4	4	205555380121_R05C01	Test/EEBB
**196**	TSC		frontal	M	0	1,5	1,5	205555380121_R06C01	Test/EEBB
**197**	TSC		temporal	M	0	3	3	205555380121_R07C01	Test/EEBB
**198**	FCD 1A		parietal	M	9.0	10.0	19.0	203723190073_R06C01	Test/ILAE
**199**	FCD 2A		temporal	M	4.0	10.0	14.0	203723190073_R03C01	Test//ILAE
**200**	FCD 2A	*DEPDC5*	frontal	M	8.0	42.0	50.0	203723190073_R04C01	Test//ILAE
**201**	FCD 2A	*MTOR*	NOS	M	0.33	1.25	1.6	203723190073_R05C01	Test//ILAE
**202**	FCD 2A	*AKT3*	frontal	M	2.5	16.5	19.0	203723190080_R01C01	Test//ILAE
**203**	FCD 2B		frontal	M	13.0	15.0	28.0	203723190073_R01C01	Test//ILAE
**204**	FCD 2B		frontal	M	13.0	12.0	25.0	203726680009_R08C01	Test//ILAE
**205**	FCD 2B		occipital	M	5.0	19.0	24.0	203723190079_R08C01	Test//ILAE
**206**	FCD 2B	*MTOR*	temporal	M	2.0	37.0	39.0	203726680023_R04C01	Test//ILAE
**207**	FCD 3D		parietal	M	0.0	37.0	37.0	203723190073_R02C01	Test//ILAE
**208**	FCD 3D		frontal	M	5.0	12.0	17.0	203723190080_R02C01	Test//ILAE
**209**	mMCD		temporal	M	17.0	10.0	27.0	203723190073_R07C01	Test//ILAE
**210**	mMCD		frontal	M	5.0	11.0	16.0	203723190073_R08C01	Test//ILAE
**211**	MOGHE	*SLC35A2*	frontal	M	0.6	7.4	8.0	203723190079_R07C01	Test//ILAE
**212**	MOGHE	*SLC35A2*	frontal	M	20.0	8.0	28.0	203726680023_R02C01	Test//ILAE
**213**	TLE/CTRL	HS	temporal	M	4.0	22.0	26.0	203723190079_R03C01	Test//ILAE
**214**	TLE/CTRL	HS	temporal	M	22.0	22.0	44.0	203723190079_R04C01	Test//ILAE
**215**	TLE/CTRL	HS	temporal	M	25.0	17.0	42.0	203723190079_R06C01	Test//ILAE

Legend to Supplement Table 1: AKT3 – actin kinase 3; AVM – arterio-venous malformation; CAV – cavernoma; CTRL – control; EEBB – European Epilepsy Brain Bank; F – female; FCD – focal cortical dysplasia; HME – hemimegalencephaly; ILAE – International League Against Epilepsy; M – male; mMCD – mild malformation of cortical development; MOGHE – mMCD with oligodendroglial hyperplasia in epilepsy; MTOR – mammalian target of rapamycin; NCx – neocortex; NA – not specified; OP - operation/surgery; PMG – polymicrogyria; RE – Rasmussen Encephalitis; SLC35A2 – solute carrier; SWS – Sturge Weber syndrome; TBI – traumatic brain injury; TLE – temporal lobe epilepsy; TSC – tuberous sclerosis complex; HS – hippocampal sclerosis
